# Nutritional Association of Quality of Life Among Colorectal Cancer Survivors in Malaysia: A 6-Month Follow-Up Study

**DOI:** 10.3390/ijerph22111648

**Published:** 2025-10-30

**Authors:** Ainaa Almardhiyah Abd Rashid, Hamid Jan Jan Mohamed, Amal K. Mitra, Lydiatul Shima Ashari, Mohd Razif Shahril, Lee Yeong Yeh, Raja Affendi Raja Ali

**Affiliations:** 1 Nutrition Program, Faculty of Food Science and Nutrition, Universiti Malaysia Sabah, Kota Kinabalu 88400, Sabah, Malaysia; ainaarashid@ums.edu.my; 2 Nutrition Program, School of Health Sciences, Universiti Sains Malaysia, Kubang Kerian 16150, Kelantan, Malaysia; lydia.shima@gmail.com; 3 Department of Public Health, Julia Jones Matthews School of Population and Public Health, Texas Tech University Health Sciences Center, Abilene, TX 79601, USA; amal.mitra@ttuhsc.edu; 4 Centre for Healthy Aging and Wellness (HCARE), Faculty of Health Sciences, Universiti Kebangsaan Malaysia, Kuala Lumpur 50300, Malaysia; razifshahril@ukm.edu.my; 5 Department of Medicine, School of Medical Sciences, Universiti Sains Malaysia, Kubang Kerian 16150, Kelantan, Malaysia; yylee@usm.my; 6 GI Function & Motility Unit, Hospital Pakar Universiti Sains Malaysia, Kubang Kerian 16150, Kelantan, Malaysia; 7 Sir Jeffrey Cheah Sunway Medical School, Faculty of Medical and Life Sciences, Sunway University, Petaling Jaya 47500, Selangor, Malaysia; affendi@sunway.edu.my

**Keywords:** colorectal cancer, health-related quality of life, nutritional parameters, follow-up study, Malaysia

## Abstract

Background: Quality of life (QoL) is a crucial outcome measure in cancer care. This study aimed to identify the association of health-related quality of life (HRQoL) among colorectal cancer (CRC) patients in Malaysia. Methods: The study was conducted from January 2021 to July 2022, recruiting CRC patients from two teaching hospitals in Malaysia. The validated Malay versions of EORTC QLQ-C30 and QLQ-CR29 questionnaires assessed physical, psychological, and social functioning. Patients were evaluated 6 months after diagnosis to determine QoL associations. Anthropometric measurements were recorded at baseline (diagnosis, 0 months) and at follow-up (6 months). Results: Among 87 CRC patients (55.2% male, mean age 59.8 ± 11.8 years), 37.9% had stage III disease and 13.8% had stage IV. Most patients (93%) underwent surgery and 78% received chemotherapy. The mean global health status score was 66.57 ± 20.75. Multiple linear regression analysis revealed that older age (*p* = 0.03), advanced cancer stage (*p* = 0.017), lower body weight (*p* = 0.017), and reduced hip circumference (*p* = 0.040) are significantly associated with poorer global health status quality of life (GHS QoL). Nutritional parameters specifically predicted functional domains: lower body weight and BMI predicted role and cognitive function, while lower hip circumference predicted emotional function. Conclusions: Advanced age, disease stage, and nutritional status are significant associations of QoL in Malaysian CRC patients. These findings highlight the importance of nutritional assessment and intervention in CRC survivorship care to optimize patient outcomes.

## 1. Introduction

According to the International Agency for Research on Cancer (IARC), which is the cancer agency of the World Health Organization, the most frequently diagnosed cancers worldwide include lung cancer, female breast cancer, and colorectal cancer (CRC) [1]. The most cancer deaths are due to lung cancer and CRC [1]. CRC is a growing burden, especially in developing countries [1].

Quality of life (QoL) has become an important outcome criterion in patients with cancer. Long-term survivors of CRC may experience persistent issues affecting their QoL [2]. Even though treatments are not curative for most patients, QoL is often considered the predictor of treatment success. As a result, the goals of treatment are oriented towards improving survival, slowing tumor progression, effectively managing symptoms, and enhancing overall QoL [3,4].

Survival rate of CRC patients is greatly dependent on the cancer stage; for example, the 5-year relative survival rate exceeds 90% for the localized stage while less than 10% for the advanced and metastatic stage of the cancer [5]. A significant number of individuals who have survived CRC often endure a substantial load of symptoms, including fatigue, bowel dysfunction, depression, and insomnia [6]. This burden of symptoms has a considerable impact on their QoL following the completion of treatment. It also has a profound influence on the overall CRC survivorship.

Despite growing recognition of QoL as a critical outcome in CRC care, there are gaps in the literature on the associations of QoL among Asian populations, particularly in Malaysia where cultural, dietary, and healthcare factors may influence outcomes differently than in Western populations. Most existing studies focus on Western cohorts, and the specific associated factors of QoL in Malaysian CRC patients remain poorly understood. Furthermore, the relationship between nutritional status and QoL in this population has not been adequately explored.

Therefore, this study aimed to identify the sociodemographic, clinicopathologic, and nutritional associations of QoL among CRC survivors at 6 months after diagnosis in Malaysia. Understanding these associations could inform targeted interventions to improve QoL and optimize survivorship care in this population.

## 2. Materials and Methods

### 2.1. Study Design, Setting, Study Participants

This follow-up study was conducted from January 2021 to July 2022 by patient selection using a convenience sampling technique, as first-come, first-served basis. Patients were recruited from all eligible CRC patients at two tertiary teaching hospitals in Malaysia, namely, Hospital Universiti Sains Malaysia (HUSM) and Hospital Canselor Tuanku Muhriz, Universiti Kebangsaan Malaysia (HCTM UKM). Anthropometric measurements were recorded at baseline (at diagnosis, 0 months), and patients were followed up at 6 months after diagnosis for reassessment of anthropometric parameters and collection of new quality of life (QoL) data.

The follow-up interval varied slightly, ranging from 6 to 7 months, as patients attended regular monthly visits for cancer treatment. Most patients initiated their cancer treatment within one month after diagnosis, as cases were promptly referred to the surgical department. The units for cancer patients at HUSM included the oncology clinic, surgery clinic, and radiology unit. The units for patient recruitment at HCTM UKM included the obesity clinic, outpatient clinic, and private clinics.

HUSM is located in the suburban city of Kota Bharu, a north-eastern region of Peninsular Malaysia, having a population density of 122 people per km^2^. HCTM UKM is located in the metropolitan city of Kuala Lumpur at Klang Valley of Peninsular Malaysia, having a population density of 8045 per km^2^. These two hospitals, HUSM and HCTM UKM, represented the suburban and urban areas, respectively, in Malaysia.

The study adheres to the Strengthening the Reporting of Observational Studies in Epidemiology (STROBE) reporting guidelines. Inclusion criteria included (1) patients between the ages of 18 and 80 years; (2) histopathologically confirmed CRC; (3) diagnosed no more than two years before study enrolment; and (4) had no previous cancer diagnosis at other anatomic sites. Exclusion criteria included (1) mental disorders, such as major depression, schizophrenia, and anxiety; (2) physical disability affecting independent self-care; and (3) females being pregnant or breastfeeding. Cancer staging was determined based on the tumor–node–metastasis (TNM) classification system according to the American Joint Committee on Cancer (AJCC).

Ethical approval was obtained from the Human Research Ethics Committee of USM (USM/JEPeM/19060354; date of approval: 3 March 2020), and the Universiti Kebangsaan Malaysia Medical and Research Ethics Committee (UKMREC; FF-2020-005). The study objectives and research procedures were explained to the potential patients, and written informed consent was obtained from each patient before enrolment.

### 2.2. Sample Size Estimation and Power

**Figure 1:**
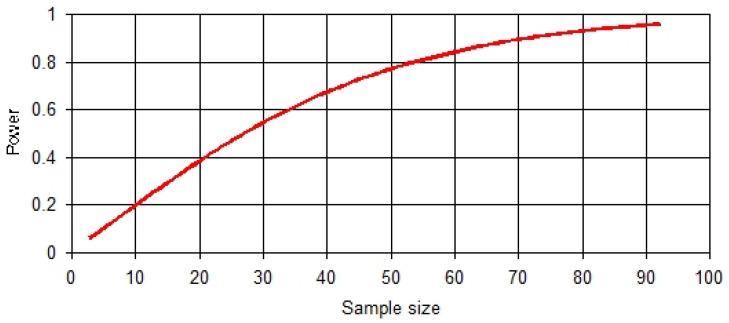
Power curve.

The sample size for the follow-up study was computed using Power and Sample software (PS software) version 3.0 by William D. Dupont and Walton D. Plummer (1997–2009). Assuming the true difference (δ) is 13, σ = 23.78, 90% power of study, alpha 0.05, and 1 to 1 ratio participation, we needed 71 subjects. With the addition of 30% non-response rate, the total sample size for the study was 92. The power curve is shown in Figure 1.

The patients were followed up for 6 months after diagnosis, at which time a proportion of them were still receiving active cancer treatment to assess their QoL in terms of daily activities, physical strength, pain, sleep, and mental health. Anthropometric assessments were conducted at baseline (0 months) and repeated at follow-up (6 months after diagnosis). Waist and hip circumferences (WC) (HC) were measured using a measuring tape (Seca, 201, Hamburg, Germany), and height was measured using a stadiometer (Seca, 217, Hamburg, Germany), and weight was measured using a body composition analyzer (TANITA SC-330, 2008).

### 2.3. Study Instruments and Administration

QoL was obtained through structured face-to-face interviews conducted on-site at the hospital during patients’ scheduled medical appointments using the Malay version of two sets of established questionnaires obtained from the European Organisation for Research and Treatment of Cancer (EORTC) [7]. The same mode and setting of data collection were used consistently for all participants at both baseline and follow-up assessments. The core questionnaire, QLQ-C30, was used to evaluate the overall health, functions, symptoms, and financial implications of the disease. This questionnaire was composed of both multi-item scales and single-item measures, ranging from ‘not at all’ to ‘very much’. The questionnaire comprised one GHS QoL scale, five functional scales, three symptom scales, and six single items.

The supplementary questionnaire module, QLQ-CR29, specifically assessed the QoL among CRC patients. It included four multi-item scales and nineteen single-items. These questions comprised information about body image, sexual function, and patients’ future perspective. In functioning scales, a high score indicated a higher level of functioning and better overall functioning. Conversely, in symptom scales, a high score indicated more severe symptoms or worse symptomatology. For sex-specific QLQ-CR29 items, including sexual interest (men), sexual interest (women), and impotence (men), responses marked as “not applicable” were coded as missing and excluded from domain scoring, following the EORTC QLQ-CR29 scoring manual. Analyses were conducted separately for male (n = 48) and female (n = 39) patients. All QLQ-C30 and QLQ-CR29 item scores were linearly transformed to a 0–100 scale, in which higher scores on functional and global health scales indicate better functioning or QoL, while higher scores on symptom scales indicate greater symptom burden, following the EORTC Quality of Life Group Scoring Manual [7].

The Malay version of QLQ-C30 had 30 questions and the Malay version of QLQ-CR29 included 29 questions with answers using a Likert scale, ranging from “not at all” to “very much”. Each response scale was recorded and transformed into a score between 0 and 100 using a description.

### 2.4. Statistical Analysis

Data analyses were conducted using IBM SPSS statistic version 22.0 (Chicago, IL, USA). The data were tested, cleaned, and explored to investigate the graphical distribution of the data by using histogram, box whisker plot, and Shapiro–Wilk to understand the data distribution. For descriptive results, numerical and continuous data with normal distribution were presented as mean and standard deviation (SD) while skewed distributed data were presented as median and interquartile range (IQR). In addition, categorical data were presented as frequency and percentage.

Multiple linear regression analyses were used to determine factors that predicted QoL, functional scales, and symptom scales for the QLQ-C30 and QLQ-CR29 questionnaires. Both numerical (age and anthropometry) and categorical (sex and CRC stages) independent variables were used in the model.

Model building strategy: We first conducted a correlation test. The variables that had a high correlation were further assessed for multicollinearity. Any independent variables with multicollinearity were restricted in the model. In the regression model, we used the variables that had a *p*-value < 0.05 or closer.

## 3. Results

### 3.1. Patient Characteristics and Treatments

The mean (SD) patient age was 59.83 (11.79) with the majority being male (55.2%). Most of the CRC patients had stage III (37.9%) cancer, followed by stage II (29.9%), stage I (14.9%), stage IV (13.8%), and unknown (3.4%). The majority of the patients underwent surgical treatment; among the surgery types, about 45% were laparoscopic anterior resections. Chemotherapy was given in 78.2% patients, and radiotherapy in 37.9% patients. In the chemotherapy/radiotherapy group, most of them had adjuvant chemotherapy (44.8%), meaning that the chemotherapy was implemented after surgery. A combination of folinic acid, fluorouracil, and oxaliplatin (FOLFOX) was the most frequent chemotherapy regime provided to the CRC patients (Table 1).

### 3.2. Assessment of Global Health Status, Functioning, and Symptoms

In general, the mean (SD) score for global health status (GHS/QoL) was 66.57 (20.75). Scores above 60 in functioning scores represented high or better functioning status. Among the functional domains, cognitive function had the highest scores (86.76 ± 19.56), whereas physical functioning had the lowest scores (65.13 ± 22.61). Of the symptom scales, fatigue was the worst common symptom, having a QoL score of 47.06 ± 23.96, followed by pain (43.68 ± 24.54), loss of appetite (39.08 ± 27.00), and nausea and vomiting (36.59 ± 25.52) (Table 2).

Most CRC patients expressed that they were satisfied with their body image as it scored the highest (85.95 ± 17.73) among the functioning scale in QLQ-C29. However, mean score for anxiety was low (57.47 ± 22.56), indicating the presence of significant anxiety among CRC patients. Sexual interest for both men and women scored the second highest among the functional domains, suggesting that intimacy and sexual well-being continue to play a meaningful role in overall quality of life despite the disease burden. The other common symptoms and their corresponding QoL scores were as follows: urinary frequency (43.29 ± 19.93), abdominal pain (22.60 ± 24.11), and stool frequency (21.26 ± 21.37) (Table 3).

### 3.3. Multiple Linear Regression Analysis for Associations of QoL

Results of multiple linear regression analysis in associating QoL in CRC patients are presented in Table 4, Table 5 and Table 6 Independent variables included in the model were sociodemographic variables (age and sex), clinicopathologic feature (stage of cancer), and changes in anthropometry, such as body weight, BMI, WC, and HC. Changes in anthropometric parameters were calculated as follow-up value minus baseline value (Δ = 6-month—baseline), expressed in kilograms (kg), kilograms per square meter (kg/m^2^), or centimeter (cm), as appropriate. Patients with missing data at either time point were excluded from the respective analysis (complete-case analysis).

Inclusion of the independent variables in the model were based on the results of the correlation test and any multicollinearity. The independent variables in the model had no multicollinearity.

More than half of the CRC patients presented with malnutrition, classified according to the WHO Asia-Pacific BMI classification, which includes both undernutrition (BMI < 18.5 kg/m^2^) and excess adiposity (BMI ≥ 23.0 kg/m^2^) as forms of nutritional imbalance.

### 3.4. Associations of QoL for Functioning Scales

Table 4 shows that older age (*p* = 0.030), advanced stage of cancer (*p* = 0.017), lower body weight (*p* = 0.017), and lower HC (*p* = 0.040) were significant associations of a lower global health status/QoL (GHS/QoL) among the patients. Older age was found to be a significant association for the worsening of physical function (*p* = 0.001), role function (*p* = 0.012), and cognitive function (*p* = 0.002). The advanced stage of CRC was another independent and significant association of role function (*p* = 0.002) and emotional function (*p* = 0.032). Among the anthropometry parameters, lower body weight significantly predicted role functioning (*p* = 0.001) and cognitive functioning (*p* = 0.001), while lower HC predicted emotional function (*p* = 0.043).

### 3.5. Associations of QoL for Physical Symptoms

Among the physical symptoms presented in Table 5, more body pain was predicted by older age (*p* = 0.006), symptoms of fatigue were predicted by lower body weight (*p* < 0.001) and low BMI (*p* = 0.003), and insomnia was significantly predicted by an advanced stage of cancer (*p* = 0.048), lower body weight (*p* = 0.041), and low BMI (*p* = 0.047).

Among other symptoms (Table 6), older age predicted urinary incontinence (*p* = 0.004) and frequent stool motions (*p* = 0.034). An advanced disease stage significantly predicted stools having blood and mucus (*p* = 0.013), buttock pain (*p* = 0.029), frequent stool motions (*p* = 0.007), fecal incontinence (*p* = 0.001), and bloating (*p* = 0.013).

## 4. Discussion

This study identified several significant associations of HRQoL among Malaysian CRC patients at 6 months after diagnosis. Advanced age, chronic disease stage, and poor nutritional status (lower body weight and hip circumference) emerged as primary determinants of reduced QoL. Notably, nutritional parameters showed domain-specific associations, with anthropometric measures differentially predicting physical, cognitive, and emotional functioning.

In the present study, although the overall GHS/QoL was somewhat high, it was poorer among patients of an older age and those with an advanced stage of CRC. These findings are consistent with another study from Malaysia which reported a poorer score of GHS/QoL among patients aged more than 60 years, and those with TNM (tumor, node, and metastases) stage IV compared with stage I to III of CRC [8]. In another study in Greece in patients with CRC, QoL was shown to significantly deteriorate from stage I to stage II and from stage II to stage III of cancer [9]. Based on these data, one may deduce a reasonable conclusion that patients with an early CRC stage, when the disease has not yet impaired their fundamental biological, physical, and health function and activities, should be expected to have a better QoL. The age-related decline in QoL likely reflects the cumulative impact of treatment toxicity, reduced physiological reserve, and increased comorbidity burden in older patients. This finding underscores the need for age-adapted survivorship care plans.

Interestingly, our study subjects had high overall QoL scores and remained satisfied with their body image. This could be attributable to a stronger social support for cancer patients (or any sick patients) which is more of a cultural norm in many developing countries. However, these patients must have gone through much pain, agony, and anxiety, as demonstrated by the deterioration of their physical function, role function, and cognitive functions in this study. Malaysian cancer patients often cope through strong family and community support, religious and spiritual practices such as prayer, and maintaining a positive outlook rooted in cultural values of patience and acceptance, which help them manage anxiety and emotional distress during recovery.

The prominent role of nutritional status in associating QoL represents a key finding of our study. Over half of our patients presented with malnutrition, which significantly impacted multiple functional domains. This contrasts with Western studies where overweight/obesity is more common. The association between hip circumference and emotional function may reflect body image concerns or indicate muscle wasting affecting psychological well-being. These findings suggest that comprehensive nutritional assessment and intervention should be integral components of CRC survivorship care in Malaysia.

Similarly to patients with other types of cancer [10], physical and role functioning were better preserved among the younger group of CRC patients compared to older CRC patients. The present study exhibited significant association between CRC stage with emotional functions, role function, and insomnia. This study’s results align with previous studies which reported emotional functions deteriorating with advanced stage of the disease [11], role functions significantly declining with increased stage of the disease [12], and insomnia being a disturbing side effect of the treatment of cancer [13]. Beyond clinical factors, psychosocial and cultural influences may shape QoL perceptions. In Malaysia, strong family ties, religious faith, and cultural values emphasizing resilience and modesty can affect how patients cope with illness and express distress. Recognizing these factors is essential for culturally sensitive and holistic survivorship care.

Patients’ poor nutritional status adversely affected their QoL, as shown consistently in our study data and in another cross-sectional Mexican study with 65 men and 48 women with CRC [14]. In both the studies, malnutrition was shown to have a profound effect on the patients’ functionality and QoL indices. However, these studies, being cross-sectional in nature, could not show any changes in the functioning and QoL of the patients over time.

In a prospective cohort study, 459 CRC survivors (stage I to II) were followed from diagnosis up to 24 months post-treatment in the Netherlands [15]. The patients’ nutritional status of this cohort was not comparable to our study patients, as 44% of the patients in the Netherlands study were overweight and 31% were obese at diagnosis, where more than half of our patients were malnourished. Still, the findings of the Netherlands study are worth mentioning because an increase in adipose tissue and muscle function of the patients were longitudinally associated with better QoL and less fatigue, regardless of pre-treatment body composition. These findings are consistent with our present study, which also observed better QoL among individuals with higher body weight and HC within a similar time frame from the diagnosis to the first two years post-treatment. Moreover, a normal BMI level was associated with better physical, role function, and social functions from 6 weeks to 24 months after CRC treatment [16], which is consistent with the present study’s findings of a positive association between BMI, fat mass, and role function.

Regarding specific symptom scale items, significant associations were documented in our study between CRC stage and buttock pain, increased stool frequency, blood and mucus in stool, and fecal incontinence. These symptoms are more frequently reported by patients with advanced TNM stages [17]. Most of our CRC patients had stage III cancer, and tumors were located in the rectosigmoid region of the colon.

### 4.1. Clinical Implications

Our findings have important clinical implications. First, routine screening for malnutrition using anthropometric measures and nutritional assessments should be integrated into oncology follow-up care to enable early identification of patients at risk of poor QoL outcomes. Second, targeted nutritional interventions, such as diet counseling, oral nutrition support, or referral to dietitians, may improve not only physical outcomes but also cognitive and emotional functioning and treatment tolerance. Third, age-stratified survivorship care programs are warranted, as younger and older survivors may differ in nutritional vulnerabilities, psychosocial challenges, and rehabilitation potential. Finally, these findings underscore the importance of multidisciplinary survivorship clinics, where nutritionists, oncologists, and mental health professionals collaborate to optimize holistic patient recovery and improve overall quality of life.

Based on the high rates of anxiety in this study’s patients, we recommend to explore coping strategies and psychological support.

### 4.2. Limitations

Several limitations warrant consideration. The study design precludes causal inferences and temporal assessment of QoL changes. The convenience sampling method may limit generalizability, and the relatively small sample size may have reduced statistical power for subgroup analyses. Additionally, we lacked baseline QoL data from diagnosis, preventing assessment of QoL trajectories, and the relatively short follow-up period limited our ability to observe long-term changes. Possible recall bias may have occurred during data collection via interviews, and the lack of multivariate analysis considering different treatment modalities and broader socioeconomic factors, such as income, education, and access to care, may have limited the depth of interpretation. Future longitudinal studies with larger, more diverse samples and baseline assessments would strengthen evidence in this area.

Another limitation was the lengthy list of EORTC QLQ C30 and QLQ-CR29, which could potentially limit the accurate responses of the study patients. Furthermore, QLQ C30 and QLQ-CR29 assessments were not conducted at diagnosis, preventing evaluation of QoL trends from diagnosis to survivorship. Thus, repeated assessments of QoL are suggested to follow patient courses of QoL [18]. Incorporating qualitative data into quantitative research could provide deeper insights into the barriers and challenges faced by patients. Collaboration with psychologists or psychiatrists may also assist in providing emotional support and motivate the patients during data collection.

Despite the limitations, one of the major strengths of the study was the use of local (Malay) versions of EORTC questionnaires which were validated in earlier studies.

## 5. Conclusions

This study demonstrates that age, disease stage, and nutritional status are significant associations of QoL in Malaysian CRC survivors. The prominent impact of malnutrition on multiple functional domains highlights the critical importance of nutritional assessment and intervention in survivorship care. Healthcare providers should implement routine nutritional screening and develop targeted interventions to optimize QoL outcomes. Future longitudinal research with larger samples and baseline assessments is needed to better understand QoL trajectories and inform evidence-based survivorship care programs for CRC patients in Malaysia and similar populations.

## Figures and Tables

**Table 1:** Baseline characteristics of CRC patients.

Variables	No (%)
Age (years), mean (SD)	59.83 (11.79)
Sex	
Male	48 (55.2)
Female	39 (44.8)
Anthropometry assessment at diagnosis, mean (SD)	
Weight, kg	62.03 (14.23)
BMI, kg/m^2^	24.17 (4.82)
WC	89.17 (13.45)
HC	99.93 (11.29)
Nutritional status categories at diagnosis	
Underweight	3 (3.4)
Normal BMI	53 (60.9)
Overweight/Obese	31 (35.5)
Anthropometry assessment at 6th follow-up, mean (SD)	
Weight, kg	63.42 (15.79)
BMI, kg/m^2^	24.83 (4.82)
WC	92.48 (13.03)
HC	102.78 (14.30)
Nutritional status categories at 6 months after diagnosis	
Underweight	4 (4.6)
Normal BMI	49 (56.3)
Overweight/Obese	34 (39.1)
Stage of CRC	
Stage I	13 (14.9)
Stage II	26 (29.9)
Stage III	33 (37.9)
Stage IV	12 (13.8)
Unknown	3 (3.4)
Tumor location site	
Ascending colon	5 (5.7)
Hepatic flexure	6 (6.9)
Splenic flexure	4 (4.6)
Sigmoid	7 (8.0)
Rectum	28 (32.2)
Rectosigmoid	32 (36.8)
No available data	5 (5.7)
Surgical types	
Laparoscopic anterior resection	39 (44.8)
Laparoscopic anterior resection + Hartmann’s procedure	4 (4.6)
Anterior resection	7 (8.0)
Laparoscopic abdominoperineal resection	8 (9.2)
Sigmoid colectomy	10 (11.5)
Others	13 (14.9)
Only underwent surgery	6 (6.9)
Chemotherapy treatment	
Yes	68 (78.2)
No	19 (21.8)
Radiotherapy treatment	
Yes	33 (37.9)
No	54 (62.1)
Chemotherapy/Radiotherapy treatment types	
Neoadjuvant chemotherapy	1 (1.1)
Adjuvant chemotherapy	39 (44.8)
Palliative chemotherapy	4 (4.6)
Neoadjuvant radiotherapy	4 (4.6)
Adjuvant radiotherapy	8 (9.2)
Neoadjuvant CCRT	12 (13.8)
CCRT	3 (3.4)
Adjuvant CCRT	6 (6.9)
Adjuvant palliative	1 (1.1)
None	9 (10.3)

**Table 2:** Mean scores of all items in QLQ-C30 of CRC patients.

Variables	Mean (SD)
Global Health Status/GHS QoL	66.57 (20.75)
Functioning scale	
Physical functioning	65.13 (22.61)
Role functioning	66.47 (25.98)
Emotional functioning	77.30 (18.96)
Cognitive functioning	86.67 (19.56)
Social functioning	69.73 (22.38)
Symptom scales	
Fatigue	47.06 (23.96)
Nausea and vomiting	36.59 (25.52)
Pain	43.68 (24.54)
Dyspnea	10.34 (18.93)
Insomnia	16.09 (24.07)
Appetite loss	39.08 (27.00)
Constipation	15.58 (21.53)
Diarrhea	13.41 (19.99)
Financial difficulties	22.73 (27.47)

**Table 3:** Mean scores of all items in QLQ-C29 of CRC patients.

Variables	Mean (SD)
Functioning scale	
Body image	85.95 (17.73)
Anxiety	57.47 (22.56)
Weight	73.95 (22.40)
Sexual interest (men)	76.39 (25.69)
Sexual interest (women)	78.63 (23.55)
Symptom scales	
Urinary frequency	43.29 (19.93)
Blood and mucus in stool	9.00 (13.88)
Stool frequency	21.26 (21.37)
Urinary incontinence	9.20 (16.81)
Dysuria	8.81 (12.15)
Abdominal pain	22.60 (24.11)
Buttock pain	12.65 (17.79)
Dry mouth	13.60 (20.89)
Hair loss	10.92 (15.21)
Taste	16.09 (21.18)
Flatulence	17.43 (19.51)
Fecal incontinence	14.75 (20.24)
Sore skin	9.96 (17.69)
Embarrassment	7.09 (11.26)
Stoma care problems	31.11 (36.66)
Impotence	7.64 (19.73)

**Table 4:** Association factors of quality of life of CRC patients for functioning scales (QLQ-C30).

Variables	*β*	95% *CI*	*p*-Value	*R*^2^ *	*β*	95% *CI*	*p*-Value	*R*^2^ *	*β*	95% *CI*	*p*-Value	*R*^2^ *
	Global health status/QoL				Physical functioning				Role functioning			
Age	−0.409	−0.778, −0.040	**0.030**	0.113	−0.673	−1.060, −0.286	**0.001**	0.107	−0.590	−1.047, −0.132	**0.012**	0.123
Sex	1.723	−7.214, 10.659	0.702		−3.109	−12.835, 6.617	0.527		7.318	−3.771, 18.408	0.193	
Stage of cancer	−5.203	−9.463, −0.943	**0.017**		−2.316	−7.091, 2.460	0.338		−8.528	−13.729, −3.326	**0.002**	
Weight changes	1.018	0.184, 1.852	**0.017**		0.129	−0.811, 1.068	0.786		1.715	0.700, 2.729	**0.001**	
BMI changes	1.092	−0.899, 3.083	0.278		−0.249	−2.433, 1.936	0.821		3.083	0.662, 5.504	**0.013**	
WC changes	0.473	−0.211, 1.158	0.173		0.395	−0.357, 1.147	0.300		−0.414	−1.272, 0.445	0.341	
HC changes	0.509	0.024, 0.994	**0.040**		0.438	−0.091, 0.967	0.104		0.421	−0.166, 1.008	0.158	
	Emotional functioning				Cognitive functioning							
Age	−0.049	−0.396, 0.297	0.778	0.059	−0.539	−0.877, −0.201	**0.002**	0.132				
Sex	−3.005	−11.156, 5.146	0.466		2.991	−5.417, 11.399	0.481					
Stage of cancer	−4.285	−8.205, −0.366	**0.032**		−3.431	−7.518, 0.655	0.099					
Weight changes	0.519	−0.261, 1.299	0.189		1.288	0.524, 2.052	**0.001**					
BMI changes	−0.021	−1.854, 1.812	0.982		2.294	0.470, 4.118	**0.014**					
WC changes	0.0451	−0.170, 1.072	0.152		0.196	−0.453, 0.845	0.549					
HC changes	0.456	0.016, 0.896	**0.043**		0.394	−0.063, 0.851	0.090					

* Adjusted *R*^2^; none of the independent variables were statistically significant (<0.05) for social functioning.

**Table 5:** Associated factors of quality of life of CRC patients for symptom scales (QLQ-C30).

Variables	*β*	95% *CI*	*p*-Value	*R*^2^ *	*β*	95% *CI*	*p*-Value	*R*^2^ *	*β*	95% *CI*	*p*-Value	*R*^2^ *
	Fatigue				Nausea and vomiting				Body Pain			
Age	0.411	−0.018, 0.840	0.060	0.115	0.241	−0.223, 0.705	0.305	0.016	0.608	0.179, 1.037	**0.006**	0.07
Sex	−9.135	−19.275, 1.006	0.077		5.716	−5.219, 16.650	0.302		−5.582	−16.095, 4.931	0.294	
Stage of cancer	4.448	−0.549, 9.444	0.080		−0.630	−6.048, 4.787	0.818		3.777	−1.370, 8.924	0.148	
Weight changes	−1.641	−2.572, −0.710	**<0.001**		0.032	−1.029, 1.093	0.952		−0.906	−1.907, 0.095	0.076	
BMI changes	−3.396	−5.592, −1.199	**0.003**		−0.017	−2.483, 2.449	0.989		−1.844	−4.182, 0.494	0.121	
WC changes	−0.406	−1.178, 0.366	0.299		−1.192	−2.004, −0.380	**0.005**		−0.029	−0.854, 0.795	0.944	
HC changes	−0.299	−0.852, 0.253	0.284		−0.147	−0.753, 0.458	0.629		−0.210	−0.783, 0.362	0.467	
	Insomnia											
Age	−0.023	−0.463, 0.418	0.919	0.034								
Sex	−2.315	−12.680, 8.050	0.658									
Stage of cancer	5.040	0.047, 10.034	**0.048**									
Weight changes	−1.017	−1.993, −0.041	**0.041**									
BMI changes	−2.304	−4.576, −0.032	**0.047**									
WC changes	−0.893	−1.654, −0.133	**0.022**									
HC changes	−0.286	−0.846, 0.275	0.313									

* Adjusted *R*^2^; none of the independent variables were statistically significant (<0.05) for dyspnea or loss of appetite.

**Table 6:** Associated factors of quality of life of CRC patients for symptom scales (QLQ-CR29).

Variables	*β*	95% *CI*	*p*-Value	*R*^2^ *	*β*	95% *CI*	*p*-Value	*R*^2^ *	*β*	95% *CI*	*p*-Value	*R*^2^ *
	Blood and mucus in stool				Urinary incontinence				Buttock pain			
Age	0.226	−0.023, 0.475	0.075	0.247	0.432	0.139, 0.725	**0.004**	0.13	−0.027	−0.353, 0.298	0.868	0.055
Sex	−8.574	−14.263, −2.884	**0.004**		−1.950	−9.186, 5.287	0.594		1.095	−6.569, 8.759	0.777	
Stage of cancer	3.639	0.799, 6.479	**0.013**		0.518	−3.050, 4.087	0.774		4.094	0.423, 7.766	**0.029**	
Weight changes	−0.536	−1.100, 0.029	0.063		−0.560	−1.248, 0.128	0.109		−0.429	−1.162, 0.305	0.248	
BMI changes	−1.088	−2.408, 0.232	0.105		−0.617	−2.236, 1.003	0.451		0.159	−1.559, 1.878	0.854	
WC changes	0.705	0.274, 1.135	**0.002**		−0.570	−1.066, −0.073	**0.025**		−0.079	−0.671, 0.513	0.792	
HC changes	−0.066	−0.388, 0.257	0.687		−0.260	−0.623, 0.104	0.159		0.037	−0.388, 0.462	0.863	
	Frequent stools				Fecal Incontinence							
Age	0.413	0.032, 0.793	**0.034**	0.04	0.184	−0.184, 0.552	0.322	0.097				
Sex	−0.588	−9.800, 8.624	0.899		−8.146	−16.694, 0.402	0.062					
Stage of cancer	6.083	1.739, 10.427	**0.007**		6.969	2.942, 10.995	**0.001**					
Weight changes	−0.734	−1.608, 0.140	0.099		−0.952	−1.768, −0.136	**0.023**					
BMI changes	−1.595	−3.631, 0.441	0.123		−2.050	−3.956, −0.145	**0.035**					
WC changes	0.605	−0.082, 1.292	0.083		0.053	−0.598, 0.704	0.872					
HC changes	−0.118	−0.606, 0.369	0.631		0.030	−0.435, 0.496	0.897					

* Adjusted *R*^2^; none of the independent variables were statistically significant (<0.05) for bloating feeling; none of the independent variables were statistically significant for functioning scale variables, such as body image and anxiety.

## Data Availability

The datasets generated and/or analyzed during the current study are not publicly available but are available from the corresponding author on reasonable request. The original data questionnaire for Quality of Life of Cancer Patients (QLQ-C30) can be requested from the following website of the EORTC: https://qol.eortc.org/questionnaires/, accessed on 20 October 2025.
